# Clinical recognition memory testing in older adults should move beyond accuracy alone

**DOI:** 10.3389/fpsyg.2026.1764573

**Published:** 2026-04-24

**Authors:** Maneesh V. Kuruvilla

**Affiliations:** 1 Tasmanian Health Service, Hobart, TAS, Australia; 2 Wicking Dementia Research and Education Centre, University of Tasmania, Hobart, TAS, Australia

**Keywords:** dementia, entorhinal cortex, episodic memory, hippocampus, perirhinal cortex

## 1 Introduction

In 1972, Endel Tulving proposed a unique type of memory that encapsulated the human experience of remembering the “what,” “where,” and “when” of episodes from a person's life (Tulving, 1972). The clinical relevance of this e*pisodic* memory has only grown since then. For example, one of the current key criteria in making a diagnosis of typical Alzheimer's disease (AD) is “an initial progressive and predominant episodic memory deficit,” (Dubois et al., 2021). In neuropsychological practice, episodic memory is commonly assessed through tasks that sample different retrieval formats, including free recall, cued recall, and recognition memory. Recognition memory testing is therefore one clinically important way of probing episodic memory (although it does not capture it exhaustively and may reflect different underlying processes). In routine assessment, clinical recognition memory testing is still typically interpreted at the level of overall accuracy: whether the patient correctly endorses old items and rejects new ones. That approach is useful, but it is often too blunt for early neurodegenerative assessment.

In this opinion piece, I argue that clinical recognition memory testing in older adults should move beyond overall accuracy alone. Specifically, standard accuracy-based recognition measures should be supplemented, where feasible, by brief subjective, process-sensitive methods that better distinguish recollection from familiarity, both explained in greater detail later on. This is not simply a theoretical preference but a clinically motivated proposal: standard delayed recognition memory can appear relatively preserved in some older adults with amnestic mild cognitive impairment (aMCI), even when recollection-based performance is already declining, and this may obscure diagnostically meaningful change in prodromal AD (Koen and Yonelinas, 2016; Barba, 1997; Anderson et al., 2008; Westerberg et al., 2006; Hudon et al., 2009; Serra et al., 2010; Koen and Yonelinas, 2014; Schoemaker et al., 2014).

Although the use of subjective judgments to investigate recognition memory processes is not new and has long been central to research on episodic memory (Goldstein et al., 2019; Russo et al., 2017a,b; van den Berg et al., 2020), these approaches have gained comparatively limited traction in the development and/or use of established neuropsychological recognition memory tests. This is surprising given that recognition memory performance can arise through different experiential routes, and reducing it to overall accuracy alone may limit diagnostic sensitivity. The aim is not to replace current tests wholesale, but to embed brief, interpretable process-sensitive additions into existing clinical recognition memory paradigms.

## 2 Why standard clinical recognition memory testing is not enough

Some of the most widely used memory tests assess both free recall and recognition memory. That distinction already has clinical value. For example, two patients who return identical, poor scores on a free recall task may have distinctly different memory profiles. A patient whose performance improves with recognition cues may show a retrieval-heavy impairment profile, whereas a patient with AD may show rapid forgetting such that recognition cues offer limited benefit. This is one reason recognition memory testing remains a routine and useful part of neuropsychological practice (Elwood, 1995).

The problem is that standard recognition memory scores generally compress performance into a single outcome, usually accuracy or discrimination, even though recognition decisions may be supported by at least two partially dissociable processes: recollection and familiarity (Yonelinas, 2002). Recognition, in other words, is not synonymous with context-free knowing. Some recognition decisions are driven by the recovery of contextual details, whereas others reflect a sense that an item was encountered in the absence of contextual retrieval. Treating all correct endorsements as equivalent risks missing clinically relevant differences in how recognition is achieved.

This distinction matters most when accuracy looks deceptively reassuring. A patient may obtain a passable delayed recognition memory score but rely predominantly on familiarity, with disproportionately weak recollection. Conversely, task formats that place heavier demands on associative or contextual retrieval may reveal problems that are not obvious on standard yes/no recognition. Recognition memory tests are also easy to integrate into both verbal and visual memory tests, which makes them a pragmatic site for improvement. The question is therefore not whether recognition testing should be used, but whether it should remain restricted to accuracy alone.

## 3 Why recollection-familiarity distinctions matter in aging and prodromal AD

The more widely held theory is that recognition memory involves a dual-process, with recollection and familiarity serving as independent processes (Yonelinas, 2002; Donaldson, 1996; Mandler, 2008; Diana et al., 2007; Yonelinas, 1994; Yonelinas et al., 2022). Recollection refers to retrieval of contextual detail, whereas familiarity refers to a sense of prior occurrence without recovery of the surrounding context. The difference between how recollection and familiarity pertain to recognition memory is best encapsulated by Mandler's (1980) influential “Butcher in the Bus” illustration, that has origins dating back to Aristotle (MacLeod, 2020).

*Consider seeing a man on a bus whom you are sure that you have seen before; you “know” him in that sense. Such a recognition is usually followed by a search process asking, in effect, where could I know him from? Who is he? The search process generates likely contexts (Do I know him from work; is he a movie star, a TV commentator, the milkman?). Eventually the search may end with the insight, that's the butcher from the supermarket!* (Mandler, 1980).

Here, the “knowing” or recognizing a familiar face without being certain where from is classified as familiarity; being able to successfully recall that the person is indeed your butcher is categorized as recollection. The distinction is clinically appealing because it offers a way of moving beyond the binary question of whether an item is recognized, toward the more diagnostically informative question of how it is recognized. In reviewing how the dual processes of recognition memory have been assessed within a clinical population, the literature provides limited, but vital evidence, regarding recollection and familiarity's impact in aging and early AD. Early work using remember/know and related process-sensitive approaches in AD and aMCI established a broadly consistent prodromal pattern: recollection is disproportionately impaired, while familiarity may appear relatively spared on conventional recognition memory measures (Barba, 1997; Anderson et al., 2008; Westerberg et al., 2006; Hudon et al., 2009; Serra et al., 2010). Several later studies have reinforced this general picture, while also showing that familiarity is not a unitary or context-free process. It can look relatively preserved for some stimulus types or task formats, such as picture-based recognition memory, even when word-based or associative recollection is already compromised (Troyer et al., 2012; Embree et al., 2012; Ally et al., 2009).

This is where the proposal becomes clinically relevant. If recollection is often the more vulnerable process in healthy aging, aMCI, and early AD, then overall delayed recognition memory accuracy may underestimate clinically meaningful memory change when the task permits performance to be supported by familiarity (Koen and Yonelinas, 2016, 2014; Schoemaker et al., 2014). More recent paradigms that constrain the contribution of recollection—using response deadlines, forced-choice with similar foils, or frequency-judgment tasks—suggest that earlier reports of spared familiarity in aMCI may in part reflect methodological insensitivity: when recollection is minimized, familiarity deficits become much more obvious (Anderson et al., 2021). Thus, task format is not a minor psychometric detail; it shapes which component of recognition memory is most visible to the clinician.

The neuroanatomical literature provides further support for a process-sensitive investigation of recognition memory. Structural MRI and prognostic studies indicate that recall and associative recognition performance (recollection-weighted) map most strongly onto hippocampal integrity, whereas item-based or familiarity-weighted measures relate more to entorhinal and perirhinal cortices and to Alzheimer's disease-signature patterns of cortical thinning (Wolk et al., 2011, 2013; Belleville et al., 2014; Schoemaker et al., 2017). At the same time, this should not be taken to imply a strict one-to-one mapping. Reviews by (Squire et al. 2007); (Wixted and Squire 2010), and (Cowell et al. 2010) caution that recognition memory phenomena are unlikely to map neatly onto isolated structures, and that both memory strength and representational complexity complicate simple anatomical assignment. The clinically useful point is therefore not a rigid localization claim, but a circuit-level one: recollection- and familiarity-weighted behaviors may show different vulnerable profiles in the medial temporal lobe as aging and AD pathology unfold.

## 4 What clinicians can realistically add now to their practice

**Figure 1:**
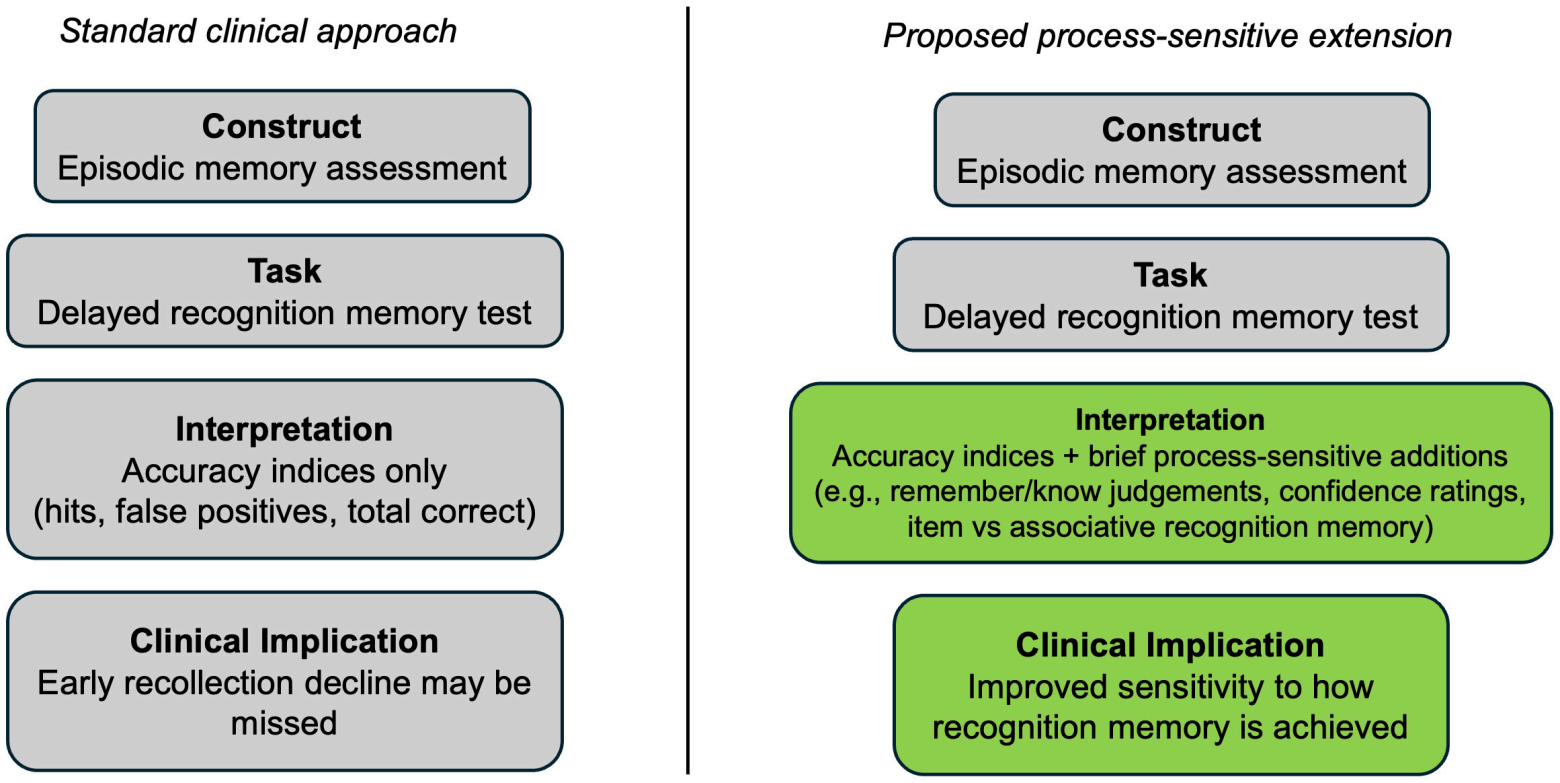
Proposed process-sensitive extension of clinical recognition memory assessment in older adults.

If this argument is to be useful for neuropsychologists, it must be operational rather than purely conceptual. The clinical takeaway from these studies is this: integrating subjective judgments into recognition memory assessments that utilize a recollection/familiarity dual-process sensitive paradigm can offer diagnostic nuance to standard recognition memory tests. The most realistic near-term goal is not to introduce complex experimental paradigms into every assessment battery, but to make modest changes to existing recognition phases (see Figure 1).

A feasible addition for routine practice is a brief remember/know judgment attached to recognized items (Tulving, 1985; Barba, 1997; Anderson et al., 2008; Westerberg et al., 2006; Hudon et al., 2009; Serra et al., 2010). After endorsing an item as old, the patient could be asked whether they “remember/are sure of it” because specific details come back to mind, or whether they simply “know/have a sense that” it was presented. This will not yield a process-pure estimate, but it can provide clinically useful qualitative and semi-quantitative information if instructions are brief and standardized. A second pragmatic option is to add confidence ratings to yes/no recognition decisions, which can later be interpreted descriptively or, in research-oriented settings, incorporated into Receiver Operating Characteristic (ROC)-type analyses (Yonelinas, 1994; Yonelinas et al., 2022). For example, patients are asked to make yes/no judgements followed by confidence ratings for each response, even ones they said no to (e.g., “on a scale of 1 (definitely old) to 6 (definitely new) do you recognize ‘word x' being presented before?”).

Task formats can also be used strategically. The manner in which tasks are administered such as forced-choice formats, picture-based recognition, time-limited decisions, frequency judgements, or item- vs. associative-recognition contrasts has the potential to differentially weight the contribution of familiarity. In clinical terms, this means that a patient who performs adequately on a standard yes/no story recognition task may nevertheless show disproportionate weakness on associative recognition or contextual/source-based formats. Conversely, preserved performance on picture-based or forced-choice recognition may reveal residual familiarity-supported capacity that is not captured by standard verbal list-learning paradigms (Koen and Yonelinas, 2014; Schoemaker et al., 2014; Troyer et al., 2012; Embree et al., 2012; Ally et al., 2009; Anderson et al., 2021; Myftaraj et al., 2025).

Not all paradigms are equally ready for routine use. Full ROC modeling and process-dissociation procedures remain best suited to research or specialist clinics because they are more assumption-laden, time-intensive, and less well-integrated into everyday neuropsychological workflows (Jacoby, 1991; Yonelinas and Jacoby, 2012). By contrast, brief remember/know judgements, confidence ratings, and deliberate contrasts between item and associative recognition are already close enough to existing practice to be piloted in routine assessment. The translational priority should therefore be brief additions that are easy to explain, score, and eventually norm.

## 5 Caveats and conclusions

A stronger clinical case for subjective and process-based measures also requires a clear acknowledgment of their limitations. Most empirical work examining recognition memory in a clinical population has been methodologically heterogenous and group-based. Subjective measures are vulnerable to response bias, and their interpretation may be affected by metacognitive demands, instruction comprehension, executive dysfunction, language impairment, education, and reduced insight. These issues are especially relevant in older adults with cognitive impairment, where a low “remember” rate could reflect true recollection failure, but also misunderstanding of instructions (e.g., “Both ‘remember' and ‘know' sound similar. How are they different?”) or broader cognitive-communication burden. For the same reason, individual-level interpretation remains difficult in the absence of robust normative anchors and standardized administration procedures.

Even so, these limitations argue for refinement rather than abandonment. The clinical challenge—and opportunity—for neuropsychologists who engage in research endeavors is to systematically and methodically embed subjective and process-based measures that are brief, interpretable, and psychometrically robust rather than replacing existing recognition memory tests wholesale. Standard recognition memory accuracy remains useful, but it is incomplete. In older adults being assessed for aMCI or prodromal AD, process-sensitive recognition memory measures may improve the detection of clinically meaningful dissociations that are otherwise obscured by overall accuracy scores. The ultimate aim should be to develop standardized paradigms and norms that facilitate individual-level decision-making in everyday clinical practice.
